# TERT promoter mutations in pancreatic endocrine tumours are rare and mainly found in tumours from patients with hereditary syndromes

**DOI:** 10.1038/srep29714

**Published:** 2016-07-14

**Authors:** João Vinagre, Joana Nabais, Jorge Pinheiro, Rui Batista, Rui Caetano Oliveira, António Pedro Gonçalves, Ana Pestana, Marta Reis, Bárbara Mesquita, Vasco Pinto, Joana Lyra, Maria Augusta Cipriano, Miguel Godinho Ferreira, José Manuel Lopes, Manuel Sobrinho-Simões, Paula Soares

**Affiliations:** 1 Instituto de Investigação e Inovação em Saúde (i3S), Universidade do Porto, Porto, 4200-135, Portugal; 2 Instituto de Patologia e Imunologia Molecular da Universidade do Porto (IPATIMUP), Porto, 4200-465, Portugal; 3 Instituto de Ciências Biomédicas Abel Salazar (ICBAS), Universidade do Porto, 4050-313, Porto, Portugal; 4 Instituto Gulbenkian de Ciência (IGC), Oeiras, 2780-156, Portugal; 5 Departmento de Patologia, Centro Hospitalar de S. João, Porto, 4200-319, Portugal; 6 Departmento de Patologia, Centro Hospitalar de Coimbra, Coimbra, 3041-801, Portugal; 7 Faculdade de Medicina da Universidade do Porto, Porto, 4200-139, Portugal

## Abstract

One of the hallmarks of cancer is its unlimited replicative potential that needs a compensatory mechanism for the consequential telomere erosion. Telomerase promoter (TERTp) mutations were recently reported as a novel mechanism for telomerase re-activation/expression in order to maintain telomere length. Pancreatic endocrine tumors (PETs) were so far recognized to rely mainly on the alternative lengthening of telomeres (ALT) mechanism. It was our objective to study if TERTp mutations were present in pancreatic endocrine tumors (PET) and could represent an alternative mechanism to ALT. TERTp mutations were detected in 7% of the cases studied and were mainly associated to patients harbouring hereditary syndromes. *In vitro*, using PET-derived cell lines and by luciferase reporter assay, these mutations confer a 2 to 4-fold increase in telomerase transcription activity. These novel alterations are able to recruit ETS transcription factor members, in particular GABP-α and ETV1, to the newly generated binding sites. We report for the first time TERTp mutations in PETs and PET-derived cell lines. Additionally, our data indicate that these mutations serve as an alternative mechanism and in an exclusive manner to ALT, in particular in patients with hereditary syndromes.

Normal somatic cells hold a limited life span due to the cell divisions they are allowed^1^. Steps towards immortalization must include deceiving the intrinsic control mechanisms that monitor telomere size. To overcome this barrier, cells must either reactivate/re-express telomerase or rely on an alternative lengthening of telomeres (ALT) mechanism. Reactivation or re-expression of telomerase is thought to be present in up to 90% of human cancers and it is generally acknowledge that proliferative cancer cells maintain their telomere length^2^. The remaining 10% to 15% of human cancers do not have detectable telomerase activity and a subset of such cases maintain telomere length relying on ALT. In sporadic PETs, at variance with the majority of other human cancers, ALT is recognized as the major mechanism for telomere elongation and mainly as a consequence of mutations in *ATRX* and *DAXX* genes^3^,^4^,^5^. Mutations in these genes are tightly associated with loss of expression of the respective proteins by immunohistochemistry and show a nearly perfect correlation with ALT phenotype^3^,^6^,^7^. Recently, two studies reported TERTp mutations in melanoma^8^,^9^. The initial proposed theoretical model of TERTp alterations presumed that the mutations lead to the creation of novel binding sites, with a consensus sequence 5′-CCCCTTCCGGG-3′, that generates a novel binding site for ETS transcription factors^8^,^9^. We and others reported the presence of these recurrent somatic mutations in a variety of human cancers and with different prevalences^10^,^11^,^12^,^13^,^14^,^15^,^16^,^17^. Although the major mechanism for telomere maintenance in PETs is ALT^3^,^6^, TERTp mutations could represent an alternative mechanism so far not described in such tumors. If so, PETs might behave similarly to what is observed in central nervous system tumors, mainly in glioblastomas, where TERTp and *ATRX* mutations are mutually exclusive, suggesting that both genetic mechanisms can confer equivalent advantages^14^. Considering this possibility, we decided to search for the occurrence of TERTp mutations in a large series of PETs (n = 55) and three PET cell lines.

## Results

### TERTp mutations, a novel contributor for PETs genetics, were mainly present in cases associated with hereditary syndromes

We detected TERTp mutations in four patients (7%), three females and one male. The association of TERTp mutation status and clinicopathological features is presented in Table 1. Three of the four (75%) TERTp mutated cases occurred in the setting of hereditary syndromes: two patients with multiple endocrine type 1 (MEN1) syndrome and a patient with Von Hippel-Lindau (VHL) syndrome (Table 2). Regarding the PET cell lines, one of the three (33%), QGP1, harboured a TERTp mutation. The mutations detected in the four PETs and in the cell line were the −124:G > A alteration.

### TERTp mutations, an alternative mechanism for telomere maintenance

**Figure 1:**
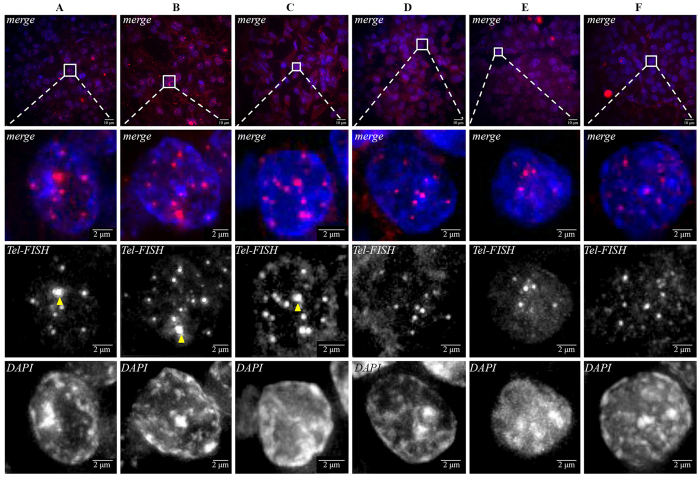
Tel-FISH in PETs. Cases depicted in panels A–C were selected due to loss of expression of ATRX and DAXX as a surrogate marker of ALT positivity; these cases presented large, ultrabright and unbalanced size telomere FISH signals (marked by the arrows), a phenotype indicative of ALT. Panels D–F, represent TERTp mutated cases 1, 3 and 4, respectively. In these cases, although some robust telomeres were present, we did not detect ultra-bright foci and the telomeres were balanced in size. One of the TERTp mutated cases was excluded from this analysis (case 2) for technical reasons, since no Tel-FISH signal was detected.

We next sought to determine if TERTp mutations could represent an alternative event to ALT. In order to exclude ALT as a relevant mechanism in the TERTp mutated PETs we studied the best-known surrogate markers: ATRX and DAXX proteins. We observed that none of the four cases with TERTp mutations had loss of expression of ATRX and DAXX (Supplementary Figure 1) thus minimizing the possibility of occurrence of ALT mechanism. The presence of ultra-bright, intra-nuclear foci of telomere FISH signals has also been used as a surrogate marker of ALT^3^,^18^; to confirm that ALT was not present in the four TERTp mutated cases we performed Tel-FISH. As a positive control for ALT telomere FISH in PETs we selected three PETs that had complete loss either of ATRX or DAXX protein expression (Fig. 1A–C). These cases presented distinctive ultra-bright foci of unbalanced size (pointed by arrows), the typical phenotype of ALT (Fig. 1A–C). In contrast, TERTp mutated cases did not present this phenotype (Fig. 1D–F).

### TERTp mutations lead to augmented telomerase transcriptional activity in PETs-derived cell lines

**Figure 2:**
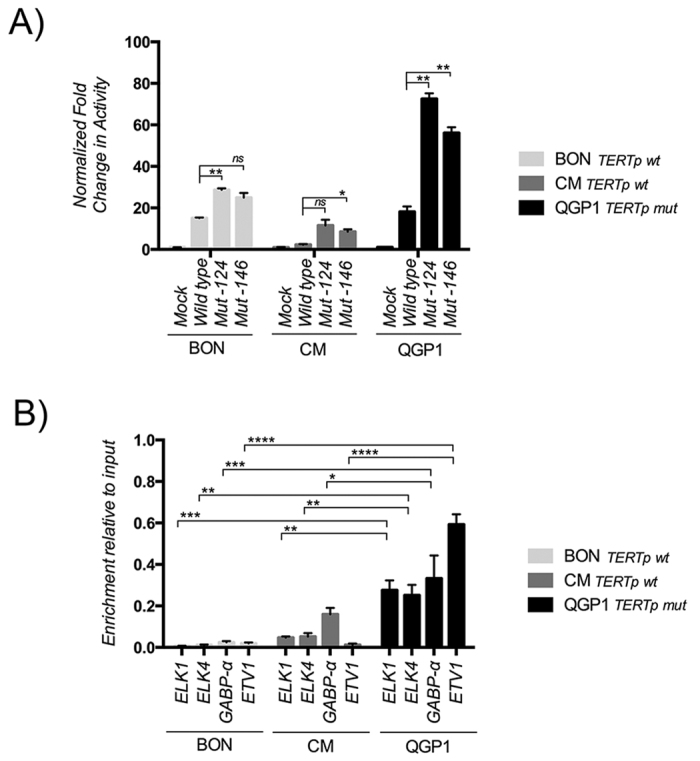
*In vitro* TERTp functional assays in the cell lines BON, CM and QGP1. (**A**) Normalized fold change in the reporter assays activity for the vectors mock, wild-type TERTp, −124 and −146 TERTp mutated vectors; Even though we only detected the −124 mutation in our samples, we also created a reporter for −146 mutation, the second most frequent alteration in other human cancers; (**B**) Quantitative analysis by RT-PCR of the ChIP revealed that QGP1 (TERTp mutated) cell line, presents significant higher amount of ETS transcription factors in comparison to BON and CM cell lines (TERTp wild-type). Additionally, to ELK1 and ELK4, we detected that GABP-α and ETV1, with the ability to bind to TERTp regions. The results are an average of at least three independent experiments. Significance levels: P < 0.0001, ****; 0.0001 < P < 0.001, ***; 0.001 < P < 0.01, **; 0.01 < P < 0.05; and P ≥ 0.05, *; and not significant (ns). Values are mean ± SEM.

Subsequent to the detection of TERTp mutation in PETs we investigated whether TERTp mutations are functional, *in vitro*, in PET-derived cell lines. By luciferase reporter assay, in comparison to the wild-type TERTp, both mutations (−124:G > A and −146:G > A) conferred an approximately 2–4 fold increased transcriptional activity in three distinct PET-derived cell lines (Fig. 2A).

### ETS family members’ transcription factors are fundamental for transcriptional activation in PETs-derived cell lines

Using JASPAR transcription factor binding profile open-access database (http://jaspardev.genereg.net/)^19^ we found that ETV1 and GABP-α were also attractive candidates since they also respond to similar consensus sequences (Supplementary Figure 2A). Following this, we took advantage of ChIP technique to test if the antibody-immunoprecipitated chromatin contained sequences of telomerase promoter. In a qualitative analysis by conventional PCR we obtained a confirmation for the presence of TERTp sequences (Supplementary Figure 2A); in order to quantify these sequences, we performed RT-PCR of the immunoprecipitates (Fig. 2B). We observed that in QGP1 (TERTp mutated) cell line, significant higher amounts of ETS-members’ transcription factors were present in comparison to BON and CM cell lines (TERTp wild-type) (Fig. 2B). Besides ELK1 and ELK4, we also detected GABP-α and ETV1, thus indicating that these transcription factors have the ability to bind to TERTp regions in PETs-derived cell lines (Fig. 2B). Finally, by EMSA with stringent probes, that avoid the native ETS in the TERTp, designed for wild-type and −124 mutated promoter sequences, we observed the presence of a shift which was only detected with the probe containing the −124 mutated sequence in the different cell lines (Supplementary Figure 2B).

### Telomere maintenance in PETs-derived cell lines

**Figure 3:**
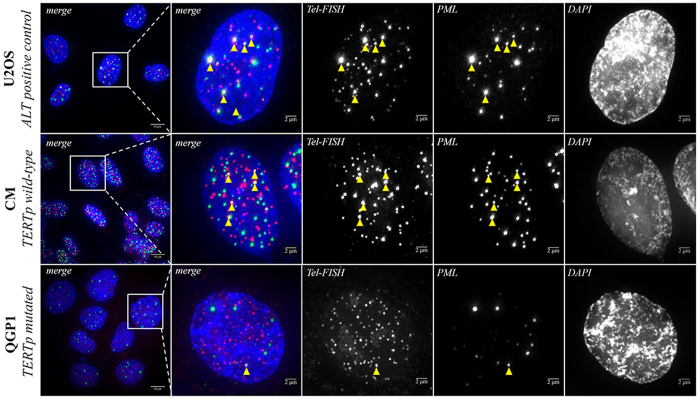
PML immunofluorescence combined with Tel-FISH in U2OS, CM and QGP cell lines. U2OS cell line represents a universal positive control for ALT mechanism. CM cell line, a TERTp wild-type cell line, presented a high co-localization of telomeric DNA with PML exhibiting an ALT positive phenotype. QGP1, the TERTp mutated cell line does not present frequent co-localization of telomeric DNA with being ALT negative. These findings recapitulate the observations in our series of PETs.

The data obtained in QGP1 experiments supported the assumption that TERTp mutations are functional and can be an alternative to ALT mechanism. So, we decided to see whether or not the cell lines recapitulate the findings in PETs. At cell level, ALT phenotype is identified by the presence of ALT-associated Promyelocytic Leukemia (PML) protein nuclear bodies that contain large amounts of telomeric DNA^20^,^21^. In Fig. 3, we observe that the positive control for ALT, U2OS cell line and CM present significantly more co-localized telomeric DNA with PML than QGP1 cell line. Furthermore, when we counted the nuclei for the presence of co-localization of Tel-FISH/PML (Supplementary Figure 3) we observed very distinctive patterns in CM and QGP cell lines. These findings indicate that QGP1 portrays an ALT negative phenotype whereas CM is ALT positive.

## Discussion

PETs had already been investigated for the presence of TERTp mutations and none were detected; however, the search was only performed in sporadic PETs^14^. In sporadic tumors *ATRX/DAXX* defects that result in ALT phenotype are present in 43–45% 4,22,23 of the cases. In contrast to this, the aforementioned ALT phenotype dropped to 6% in a subset of PETs from MEN1 syndrome patients^6^, thus leaving space for other putative mechanisms. None of the 4 cases of our series with TERTp mutations revealed loss of expression for ATRX or DAXX, a strong indication that in these tumors *ATRX/DAXX* genes do not seem to have mutations concomitantly with the TERTp alteration. Ideally, it would be necessary to genotype the *ATRX* and *DAXX* genes; however, due to the large transcript size of both genes and the absence of frozen tissue, it was not possible to follow this strategy. Furthermore, several studies have demonstrated a high correlation between the presence of mutation of *ATRX/DAXX* and loss of expression of the respective proteins^3^,^4^,^5^,^6^ thus allowing us to use protein expression as a surrogate marker for mutation presence. The detection of ATRX and DAXX proteins, excluding mutations in these genes, is also in agreement with the lack of ALT phenotype detection as observed in the Tel-FISH analysis. Altogether, these results fit with previous findings highlighting a duality of the genetic background in sporadic and hereditary PETs^3^,^4^,^6^,^22^ and present for the first time TERTp mutations as an alternative mechanism in PETs. Our results support also the assumption that TERTp mutations may play a role in hereditary PETs and that TERTp mutations and ALT are mutually exclusive, a feature observed also in central nervous system tumors^14^. *In vitro*, TERTp mutations by luciferase reporter assay in the cell lines BON, CM and QGP1 presented a 2–4 fold increased, a value consistent with previous reports^8^,^9^. Even though we only detected the −124 mutation in our samples, we created a reporter for −146 mutation, the second most frequent alteration in other human cancers, that also presented increased transcription although with less activity than the −124 mutation. Until recently, ELK1 and ELK4 were pointed as the main transcriptions factors that would bind to the newly created binding consensus induced by the mutations. Additionally, we studied GABP-α because: Bell *et al*. reported GABP-α to be an important transcription factor being able to recruit proximal ETS motifs^24^; Stern *et al*. demonstrated that TERTp mutation presents a mark of active chromatin and recruit GABP-α^25^; and Makowski *et al*. revealed that the recruitment of GABP-α is enable by the spatial architecture of native and the newly generated motifs in the TERTp region^26^. Overall, GABP-α allows the potentiation of TERTp activation^24^,^25^,^26^. Taking this into consideration, we decided to address ELK1, ELK4 and GABP-α and we included ETV1 based on binding consensus similarities and the fact that these transcription factors were expressed in the PET-derived cell lines. All the transcription factors were detected in a qualitative analysis by PCR amplification of the ChIP precipitates. Initially, we evaluated the ChIP qualitatively by PCR and we observed that the transcription factors were precipitating TERTp sequences in all the cell lines. At first glance, this result was intriguing but it is explained by the abundance of native ETS transcription factors binding sites in the telomerase core promoter^24^,^27^; there are at least 3 native ETS binding sites in the proximity in positions −91 bps, −93 bps and −190 bps upstream the ATG start site and flank the mutations around a 30 bps distance^24^. Quantitative analysis by RT-PCR of the ChIP revealed that QGP1 (TERTp mutated) cell line, presents significant higher amount of ETS transcription factors in comparison to BON and CM cell lines (TERTp wild-type). Remarkably, ETV1 and GABP-α immunoprecipitates presented higher abundance than ELK1 and ELK4 in the mutated cell line. GABP-α findings are in agreement with the three studies published recently that have pointed out GABP-α as the critical ETS transcription factor in a TERTp mutation context, being able to recruit proximal ETS motifs and potentiating TERTp activation in a mutant-specific manner^24^,^25^,^26^. Contrarily to GABP-α, ETV1 results are more difficult to interpret at this moment. Finally, we observed that cell lines recapitulate the findings in PETs; the cell line QGP1 harbouring a TERTp mutations did not present an ALT phenotype once again pointing out TERTp mutations as an alternative mechanism and in an exclusive manner. The finding that QGP1 is ALT negative concurs with novel data obtained by whole-exome sequencing that revealed that this cell line does not have mutations in *ATRX* and *DAXX*^28^.

Overall, we report for the first time TERTp mutations in PETs and PET’s-derived cell lines. TERTp mutations are noticeably prevalent in PET cases with a hereditary component. Our data indicates that TERTp mutations are functional and serves as an alternative and mutually exclusive mechanism to ALT in hereditary PETs. Previous studies reported that the prevalence of ATRX/DAXX defects were “late” events, associated with higher stage tumors and increased size^4^,^6^,^23^. The suggestion that ATRX/DAXX defects leading to ALT occurs only in a later stage is compatible with the fact that in hereditary-associated tumors there is an anticipation in the manifestation of the neoplasia. Therefore, TERTp mutations could provide the additional growth advantage that would allow the growth beyond the microadenoma size without the need for ALT. Further studies are still necessary to clarify the role played by the different mechanisms for telomere maintenance in sporadic and hereditary PETs.

## Methods

### Tissues, patient characteristics, and follow-up data

Formalin-fixed and paraffin-embedded (FFPE) tumors were retrieved from the Pathology departments of Centro Hospitalar São João and Centro Hospitalar e Universitário de Coimbra. Clinical and follow-up data were obtained by contacting the patients’ general practitioners and in-hospital registries databases. All the tumors were re-evaluated and classified according to the ENETS^29^ and the UICC/AJCC^30^,^31^ guidelines by the same pathologist (JML). A total of 55 pancreatic endocrine tumors (PETs) of 33 female patients and 22 male patients, with a mean age of 54 years (range 14–75 years) were studied. Two patients were submitted to surgical open biopsy and neoadjuvant therapy, 15 underwent enucleation, 14 were submitted to cephalic pancreaticoduodenectomy and 24 to distal pancreatectomy. The mean size of the tumors was 33 mm (range 5–100 mm); 23 tumors were located in the head, 9 in the body, 22 in the tail of the pancreas and 1 was of uncertain location. Three out 55 cases were confirmed to present a hereditary syndrome association. The majority (96%) were well-differentiated endocrine tumors, and 67% were NET G1, with less than 2 mitoses per 10 high power-fields and a Ki67 index below 2%. Extrapancreatic extension was observed in 33% of the tumors; 35% had nodal metastases; 11% had evidence of distant metastases at the time of the diagnosis. Follow-up data were obtained from 49 patients, with a mean follow up time of 61 months (range 1–182 months). Relapse or disease progression was observed in 20% patients (n = 10) with a disease-related death rate of 8% (n = 4). All the procedures described in this study were in accordance with national and institutional ethical standards. According to Portuguese law, informed consent is not required for retrospective studies.

### Cell lines

The PET cell lines used in this study corresponded to BON, CM, QGP1 and U2OS. The BON-1 cell line was cultured in 1:1 mixture of DMEM and F-12 glutamax mediums (Gibco, Massachusetts, USA). The CM and QGP1 were cultured in RPMI glutamax (Gibco). The U2OS was cultured in DMEM medium (Gibco). All the mediums were prepared with 10% fetal bovine serum (Gibco), 1% PenStrep (Gibco) and 0.5% Fungizone (Gibco).

### DNA extraction, PCR amplification and genotyping

DNA from FFPE tissues was retrieved from 10 um cuts after careful microdissection. DNA from cell lines was obtained from cell pellets. DNA extraction from FFPE and cell lines was performed using the Ultraprep Tissue DNA Kit (AHN Biotechnologie, Nordhausen, Germany) following the manufacturer’s instructions. To screen TERTp mutations, we analyzed the region containing the −124 and −146 hotspots by PCR and followed by Sanger sequencing. TERTp mutation analysis was performed with the pair of primers: Fw: 5′-CAGCGCTGCCTGAAACTC-3′ and Rv: 5′-GTCCTGCCCCTTCACCTT-3′. Amplification of genomic DNA (25–100 ng) was performed by PCR using the Qiagen Multiplex PCR kit (Qiagen, Hilden, Germany) and according to the manufacturer
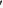
s instructions with Q solution (Qiagen). Sequencing reaction was performed with the ABI Prism BigDye Terminator Kit (Perkin-Elmer, California, USA) and the fragments were run in an ABI prism 3100 Genetic Analyzer (Perkin-Elmer). The sequencing reaction was performed in a forward direction, and an independent PCR amplification/sequencing, both in a forward and reverse direction, was performed in positive samples or samples that were inconclusive in the first amplification.

### ATRX and DAXX immunohistochemistry (IHC)

IHC for ATRX and DAXX was performed in representative tumor tissue sections previously selected by a Pathologist. Deparaffinized and rehydrated sections were subjected to antigen retrieval treatment in a pressure cooker in 10 mM sodium citrate buffer pH 6.0 for 5 minutes. The sections were incubated one hour in a humidified chamber with the primary antibodies ATRX (1:350, HPA001906) and DAXX (1:75, HPA008736) both from Sigma-Aldrich (Missouri, USA). Following the secondary antibody, the detection was obtained with a labelled streptavidin–biotin immunoperoxidase detection system (Thermo Scientific/Lab Vision, Fremont, USA) and the immunohistochemical staining was developed with 3,3′-diaminobenzidine substrate. Omission of the primary antibody incubation was used as negative control. Previously tested samples of normal pancreas were used as positive control.

### Telomere Fluorescence *In Situ* Hybridization (Tel-FISH)

FFPE sections were incubated in HistoClear II (National Diagnostics, USA), 100% (twice) at room temperature and 100% ethanol at −20 °C, for 10 minutes. Whole tissue slides were air-dried for a minimum of 30 minutes. For cultured cells, following fixation we performed an incubation 1:100 of the PML antibody (PG-M3, Santa Cruz Biotechnology). Subsequent incubation with the secondary antibody, cells were washed with PBS, three times 10 minutes. Cells were then fixed in 10% formalin for 20 minutes in the dark followed by two washes for 5 minutes in 0.05% Tween-20 in PBS. Cells were air-dried for a minimum of 30 minutes. Cells and FFPE tissue sections were denatured for 10 min in an oven (Memmert GmbH) at 80 °C in hybridization buffer (70% formamide, 25 mM MgCl2, 1 M Tris pH 7.2, 5% blocking reagent (Roche, Basel, Switzerland) containing 2.5 g/ml Cye-3-labelled telomere specific (CCCTAA) peptide nuclei acid probe (Panagene, Daejeon, Korea), followed by hybridization for 2 h at room temperature in a humid-chamber in the dark. Slides were washed with 70% formamide in 2x SSC for 10 minutes, followed by 10 minutes wash with 2x SSC (twice). Sections were incubated with DAPI (Sigma-Aldrich), mounted and imaged on an Olympus Applied Precision DeltaVision core microscope with a camera Photometrics Cascade II 1024 EM-CCD. Z stacking was performed (20 nm optical slices with x100 objective) followed by Applied Precision software SoftWorx deconvolution.

### Luciferase Promoter Reporter Assay

Briefly, a wild-type TERTp region comprising the genomic area from −290 to the −47 bps from the initial codon was amplified by PCR using the thyroid cell line XTC-1 as the DNA template. The PCR product was then cloned into pGEM-T Easy vector (Promega, Wisconsin, USA). The plasmid generated was then digested with XhoI and KpnI enzymes (Fermentas, Massachusetts, USA) and the obtained insert was subcloned into the pGL3 luciferase expression vector (Promega), creating the TERTp wild type vector. Site directed mutagenesis was performed with QuickChange Lightning Site-Directed Mutagenesis Kit (Agilent Technologies, California, USA) and was used to generate the mutations (−124 and −146 G > A) from the wild type promoter. The final vectors, pGL3-TERTp wt, −124 and −146, were generated as reporter constructs containing the firefly luciferase-encoding gene under the control of the wild type, −124 and −146 TERTp DNA motifs, respectively. Primers are available upon request. For the luciferase promoter assay, BON, CM and QGP1 cell lines were grown to 80% confluence, and transfected with 1 ug of the previously generated vectors (pGL3: wt, −124 and −146). Additionally, for normalization purposes, cells were co-transfected with 0.125 ug of pRL vector, Renilla luciferase. The transfection was performed with Lipofectamine 2000 (Lifetechnologies, Massachusetts, USA) in 12-well plates and accordingly to the manufacturer instructions. After 48 hours, the transfected cells were washed in a saline solution and stored at −80 °C for cell lysis. The remaining part of the assay was performed with the steadylite plus reporter gene assay system (PerkinElmer) and in accordance with the manufacturer instructions. Finally, the light counts from luciferase and renilla were obtained in a MicroBeta TriLux 1450 bioluminescence counter (PerkinElmer).

### Chromatin immunoprecipitation (ChIP) assay and Real Time PCR (RT-PCR)

ChIP experiments were performed using the SimpleChIP Enzymatic Chromatin IP Kit (Cell Signalling Technology, Massachusetts, USA) according to the instructions provided by the manufacturer. Briefly, BON, CM and QGP1 cells were grown until 90% confluence was reached and DNA-protein crosslinking was achieved by adding 1% formaldehyde directly in the culture medium. Digestion and isolation of the nuclei was accomplished after incubating the samples with 5 ul of micrococcal nuclease for 20 minutes at 37 °C. The quality of the chromatin preparations was assessed by electrophoresis in an agarose gel and only samples showing a pattern of chromatin fragments ranging 100–1000 bps were used in the following steps: up to 5 ug of chromatin preparation was immunoprecipitated using 1 to 2 ug of the indicated antibodies and protein G magnetic beads. DNA was then eluted from the antibody/protein G beads, purified using spin columns and used as a template for PCR and RT-PCR experiments. PCR evaluation of eluted products was performed with the conditions described above. RT-PCR employed a Sybr Fast Master Mix (KAPA Biosystems Massachusetts, USA) with a program that consisted of 45 cycles of 30 seconds at 95 °C and 30 seconds at 62 °C. Aliquots of chromatin that were not immunoprecipitated (referred as “input”) were used to normalize the results, that was calculated using the following formula: 2^−(∆Ct)^, where Ct = Ct TERTp immunoprecipitated − Ct TERTp input.

### Electrophoretic Mobility Shift Assay (EMSA)

Nuclear extracts of BON, CM AND QGP1 cells were obtained using the NE-PER Nuclear and Cytoplasmic Extraction Reagents Kit (Thermo Scientific, Massachusetts, USA), according to the instructions provided by the manufacturer, and the concentration of protein quantified using Bradford’s modified protein assay (Bio-Rad, California, USA). DNA oligonucleotides corresponding to the −124 and −146 regions of the TERTp were synthesized and labelled with biotin using the Biotin 3′ End DNA Labelling Kit (Thermo Scientific). The probes sequences are available upon request. The complementary oligonucleotides were annealed using a thermocycler and the following program: 1 cycle of 60 minutes at 22 °C, 1 cycle of 5 minutes at 95 °C, 20 cycles of 1-minute beginning at 95 °C followed by a 1 °C decrease per cycle, 1 cycle of 30 minutes at 75 °C and 40 cycles of 1 min beginning at 75 °C followed by a 1 °C decrease per cycle. EMSA binding reactions were prepared in H_2_O using the reagents contained in the LightShift Chemiluminescent EMSA Kit (Thermo Scientific) and consisted of 1x binding buffer, 1 ug/ul poly (dI.dC), 2.5% glycerol, 0.05% NP-40, 100 mM KCl, 2.5 mM MgCl2, 1 mM EDTA, 5 ug of protein nuclear extract and 20 fmol of biotin-labelled oligonucleotide (when indicated, 4000 fmol unlabelled oligonucleotide was also added as a competitive negative control). Binding reactions were incubated for 30 minutes at room temperature after which loading buffer was added. Samples were run on a 6% non-denaturing polyacrylamide gel, transferred to a nylon membrane and cross-linked in a UV chamber for 15 minutes. The membrane was developed in X-ray film using the Chemiluminescent Nucleic Acid Detection Module (Thermo Scientific) according to the instructions provided by the manufacturer.

### Statistical analysis

Statistical analysis was conducted using GraphPad Prism version 6.0 f for Mac OS X (GraphPad Software, California, USA). The results are expressed as mean ± standard error of the mean (SEM). For the analysis of the luciferase activity and the relationship between the different reporter vectors we used a two-tailed paired t-test. Two-way ANOVA was used to compare the differences between the cell lines and the abundance of transcription factors precipitated and evaluated by RT-PCR. Results were considered statistically significant whenever *P* < 0.05. As a GraphPad software default the values of statistical significance are represented as: *P* < 0.0001, ****; 0.0001 < *P* < 0.001, ***; 0.001 < *P* < 0.01, **; 0.01 < *P* < 0.05; and *P* ≥ 0.05 as not significant (NS).

## Additional Information

**How to cite this article**: Vinagre, J. *et al*. TERT promoter mutations in pancreatic endocrine tumours are rare and mainly found in tumours from patients with hereditary syndromes. *Sci. Rep*. **6**, 29714; doi: 10.1038/srep29714 (2016).

## Supplementary Material

Supplementary Information

## Figures and Tables

**Table 1:** Clinicopathological information of the patients included in this study according to the TERT promoter genotype.

	*TERT promoter genotype*	
	*wild-type*	*mutated*
Cases studied (n, %)	51 (93%)	4 (7%)
Age (range)	54 (14–75)	44 (32–55)
**Location (n, %)**		
*Head*	23 (45%)	—
*Body*	7 (14%)	2 (50%)
*Tail*	20 (39%)	2 (50%)
*n.d*.	1 (2%)	—
**Grade (n, %)**		
*G1*	35 (69%)	2 (50%)
*G2*	14 (27%)	2 (50%)
*G3*	2 (4%)	—
**pT stage (ENETS) (n, %)**		
*T1*	16 (31%)	—
*T2*	20 (39%)	2 (50%)
*T3*	12 (24%)	2 (50%)
*T4*	3 (6%)	—
**pT stage (UICC/AJCC) (n, %)**		
*T1*	18 (35%)	—
*T2*	18 (35%)	1 (25%)
*T3*	15 (30%)	3 (75%)
*T4*	—	—
Lymph node metastasis (n, %)	17 out of 31 (55%)	3 (75%)
Distant metastasis (n, %)	5 (10%)^1^	1 (25%)^2^
Hereditary syndrome association	—	3 (75%)^3^

^n.d^Not determined.

^1^All cases with liver metastasis, one of them with bone metastasis at the time of diagnosis.

^2^Liver metastasis at the time of diagnosis.

^3^Two MEN cases and one VHL.

**Table 2:** Clinicopathological and molecular relevant data of the patients with PETs harboring TERT promoter mutations.

Case number	Gender	Age^1^	Location	PET type	Size^2^	Microadenomas	Functional status	Germline mutations	pT^3^	pT^4^	Lymph node metastasis	Distant metastasis	Follow-up^5^	Status at last follow-up
*1*	F	39	body	NET G1	44	yes	insulinoma	MEN1 p.Q453X	3	3	N_1_	M_0_	107	AWD
*2*	F	55	body	NET G2	30	no	non functional	-^6^	2	2	N_1_	M_0_	9	DOD
*3*	M	51	tail	NET G1	30	yes	insulinoma	MEN1 p.A572V	2	3	N_x_	M_0_	124	DOC
*4*	F	32	tail	NET G2	94	yes	non functional	VHL p.S65W	3	3	N_x_	M_1_ (liver)	46	DOD

^1^years;

^2^mm;

^3^according to ENETS classification;

^4^according to UICC/AJCC classification; ^5^months; ^6^No *MEN1* or *VHL* mutations were detected.

AWD – alive without disease; DOD – death of disease; DOC – death of other causes. Clinical presentations: **Case 1:** Primary hyperparathyroidism and insulinoma. Known family history, both the father and a sister with pancreatic tumour, a pituitary adenoma with prolactin production and primary hyperparathyroidism; **Case 3:** Recurrent episodes of hypoglycaemia, associated with insulinoma. No other crises following surgery. Posterior history of recurrent upper gastrointestinal haemorrhage associated with gastric ulcers. The presence of gastrinoma has never been confirmed. In both ***MEN1*** cases there was no clinical or laboratorial evidence of other functioning-type NET. **Case 4:** bilateral retinal angiomatosis, cervical spinal hemangioblastoma, endolymphatic sac tumour, hepatic haemangioma and multiple renal cysts.
