# Supplementary material for: TERT promoter mutations in pancreatic endocrine tumours are rare and mainly found in tumours from patients with hereditary syndromes
Source: Sci Rep. 2016 Jul 14;6:29714. doi: 10.1038/srep29714 (PMC4944231; doi:10.1038/srep29714)
Supplement: Supplementary Information [file srep29714-s1.doc]

Supplementary Material for:

**TERT promoter mutations in pancreatic endocrine tumours are rare and mainly found in tumours from patients with hereditary syndromes**

**João Vinagre^1^**^,2,3,+^, **Joana Nabais**^4,+^, **Jorge Pinheiro**^5,+^, **Rui Batista^1^**^,2^, **Rui Oliveira**^6^, **António Pedro Gonçalves**^1,2^, **Ana Pestana**^1,2^, **Marta Reis**^1,2^, **Bárbara Mesquita**^1,2^, **Vasco Pinto**^1,2^, **Joana Lyra**^1,2^, **Maria Augusta Cipriano**^6^, **Miguel Ferreira Godinho**^4^, **José Manuel Lopes**^1,2,5,7^, **Manuel Sobrinho-Simões**^1,2,5,7^ and **Paula Soares**^1,2,7,*^.

^1^ Instituto de Investigação e Inovação em Saúde (i3S), Universidade do Porto, Porto, 4200-135, Portugal

^2^ Instituto de Patologia e Imunologia Molecular da Universidade do Porto (IPATIMUP), Porto, 4200-465, Portugal

^3^ Instituto de Ciências Biomédicas Abel Salazar (ICBAS), Universidade do Porto, 4050-313, Porto, Portugal

^4^ Instituto Gulbenkian de Ciência (IGC), Oeiras, 2780-156, Portugal

^5^ Departmento de Patologia, Centro Hospitalar de S. João, Porto, 4200-319, Portugal

^6^ Departmento de Patologia, Centro Hospitalar de Coimbra, Coimbra, 3041-801, Portugal

^7^ Faculdade de Medicina da Universidade do Porto, Porto, 4200-139, Portugal

*psoares@ipatimup.pt

+these authors contributed equally to this work

**
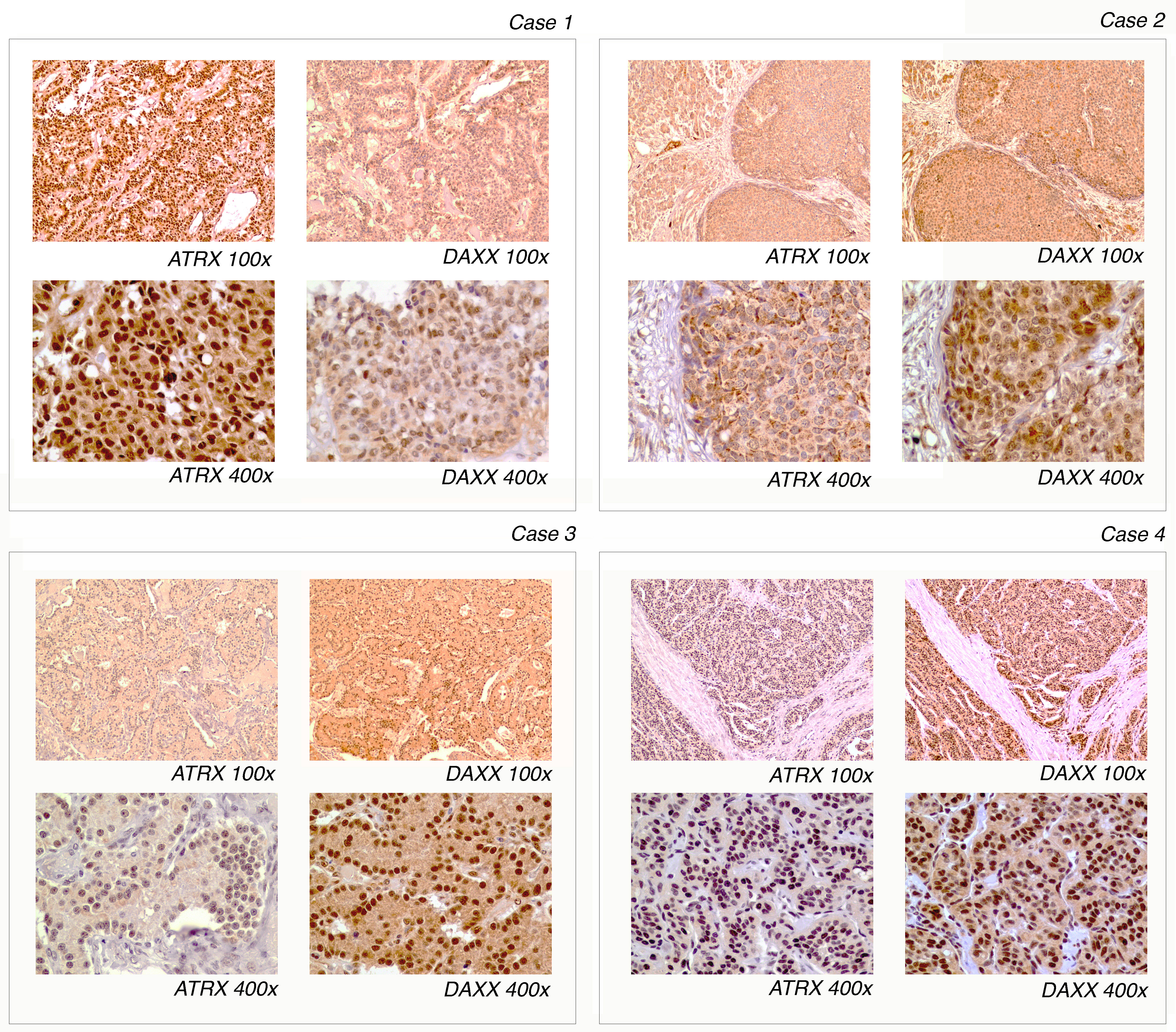
**

Supplementary figure 1

Immunohistochemistry for ATRX and DAXX proteins in cases with TERTp mutations. None of the 4 cases with TERTp mutations revealed loss of expression for ATRX or DAXX.


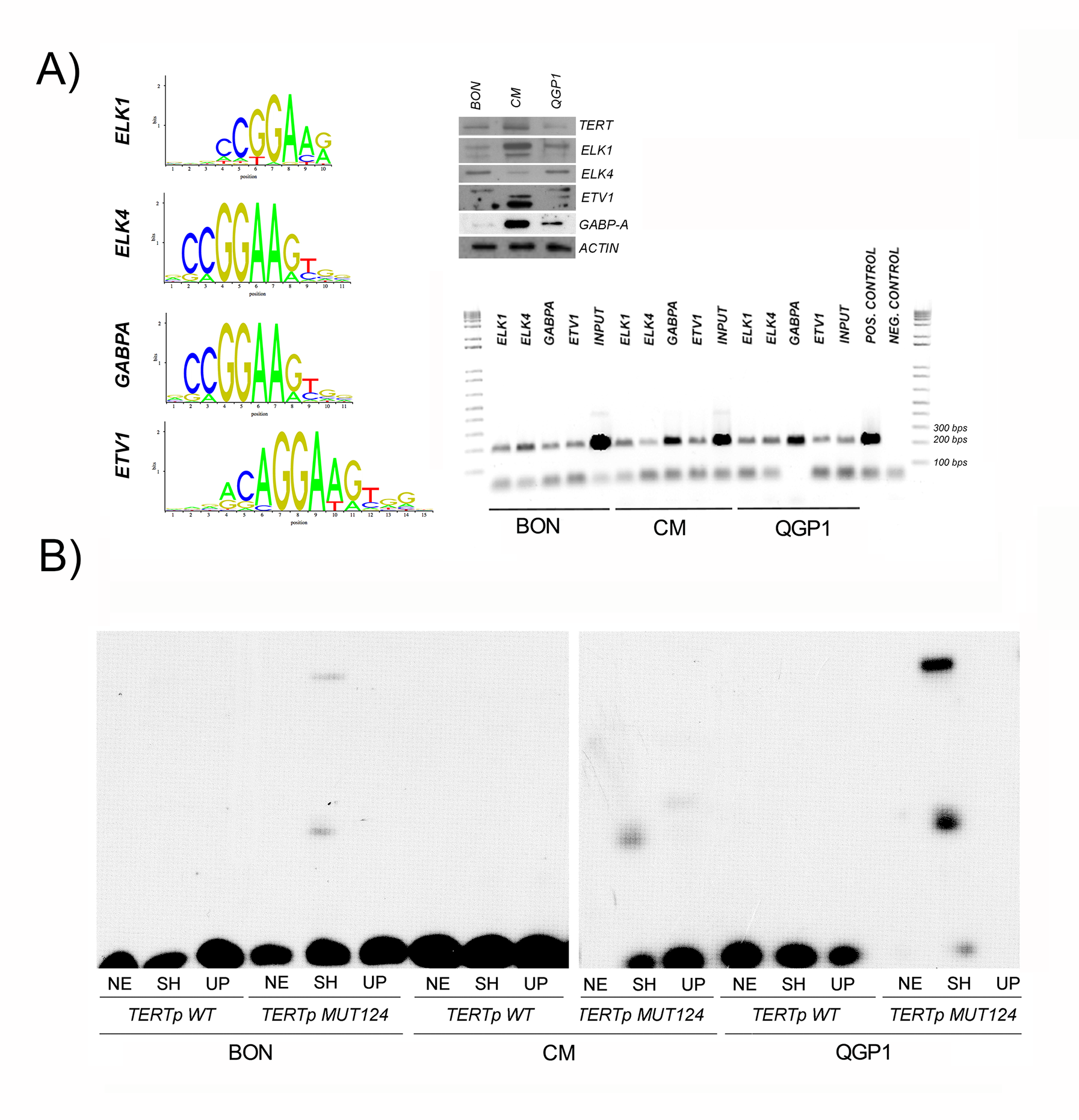


Supplementary figure 2

*In vitro* TERTp functional assays in the cell lines BON, CM and QGP1: A) JASPAR maps corresponding to the binding consensus of the transcription factor studied, ELK1, ELK4, GABP-α and ETV1; These were found to be expressed in the studied cell lines (Western-blot) as well as telomerase; All the transcription factors were detected in a qualitative analysis by PCR amplification of the ChIP precipitates; Initially, we evaluated the ChIP qualitatively by PCR and we observed that the transcription factors were precipitating TERTp sequences in all the cell lines. B) EMSA assay; Stringent probes for the mutant and wild-type promoter only presented a shift with the -124 mutated sequence probe in the different cell lines; NE – no nuclear extract, SH – nuclear extract in the presence of labelled probes for shift experiments, UP – unlabelled probe in excess as an inhibition competitor.


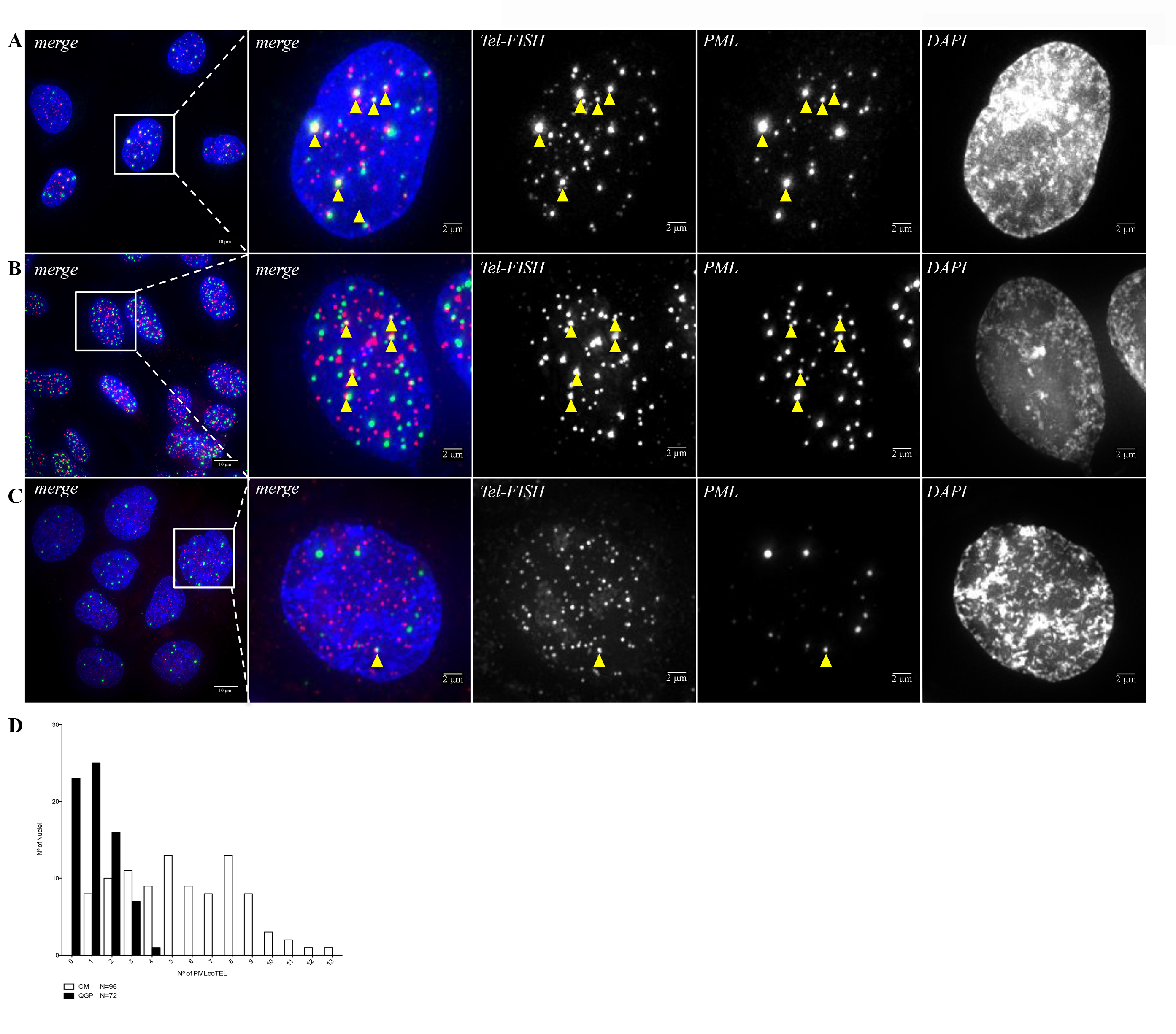


Supplementary figure 3

Co-localization of telomeric DNA with PML. Number of nuclei of CM and QGP1 cell lines with co-localized telomeric DNA and PML. The CM cell line presented more nuclei with co-localization telomeric DNA foci and PML proteins, a feature of ALT, then the QGP1 cell line. We excluded BON cell line since it had been previously characterized as an ALT negative cell line.
